# Automated Determination of Ammonium at Nanomolar Levels in Seawater by Coupling Lab-in-Syringe with Highly Sensitive Light-Emitting-Diode-Induced Fluorescence Detection

**DOI:** 10.3390/molecules30061288

**Published:** 2025-03-13

**Authors:** Xiaochen Guo, Hongliang Li, Yue Shen, Yangyang Lu, Yong Zhu, Jianfang Chen

**Affiliations:** Key Laboratory of Marine Ecosystem Dynamics, Second Institute of Oceanography, Ministry of Natural Resources, Hangzhou 310012, China; guoxcnina@163.com (X.G.); lihongliang@sio.org.cn (H.L.); sheny0501@163.com (Y.S.); luyangyang@sio.org.cn (Y.L.)

**Keywords:** ammonium, fluorometric determination, light-emitting-diode-induced fluorescence detector, lab-in-syringe, seawater

## Abstract

Ammonium concentrations in marine environments are typically found at the nanomolar level, and due to the transformation tendencies of ammonium species, there is a growing demand for a simple, convenient, and highly sensitive automated method for seawater ammonium quantification. Such a method should be suitable for in situ applications without the need for additional enrichment or extraction steps. To meet this need, we developed a highly sensitive automated flow system that integrates a portable LED-induced fluorescence detector, incorporating the novel *AccuOpt* 2000 photodetector and lab-in-syringe technology, enabling direct fluorescence measurement of trace ammonium in seawater. Key system parameters were optimized, and the seawater matrix effects were assessed. The system achieved a detection limit of 0.90 nmol/L, with a linear range up to 400 nmol/L and relative standard deviations of 0.94% (100 nmol/L, *n* = 21). The sensitivity was nearly ten-fold higher than those of conventional approaches. Seawater matrix effects, including carryover, were negligible. The system’s measurements correlated well with the indophenol blue spectrophotometric method. These results underscore the system’s strong potential for in situ/on-site monitoring of trace ammonium levels in marine environments.

## 1. Introduction

Ammonium, which includes the combined concentrations of ammonium ions (NH_4_^+^) and non-ionized ammonia molecules (NH_3_), is a highly bioavailable source of fixed nitrogen for bacterioplankton and phytoplankton. It also serves as an important indicator of water quality in marine ecosystems [1]. Ammonium plays a crucial role in the marine nitrogen cycle and is involved in various ecological processes such as biogeochemical cycling, eutrophication, algae bloom formation, acidification, and primary production [2]. As the most reduced form of inorganic nitrogen in seawater, ammonium participates in several nitrogen cycle processes, including ammonium assimilation and regeneration, dinitrogen fixation, nitrification, ammonia oxidation, and anammox. The balance between ammonium assimilation by phytoplankton and its oxidation by microbial nitrifiers is a key determinant of the nitrogen pool composition in the upper ocean [3]. Recent studies on ammonium dynamics in the ocean have highlighted its role in generating nitrous oxide, which influences the global climate system [4]. Excessive ammonium discharges into aquatic environments can have severe ecological consequences. High ammonium concentrations, particularly in its toxic ammonia form, are harmful to aquatic organisms during their larval and juvenile stages [5,6]. Ammonium pollution not only degrades water quality and disrupts aquatic habitats but also increases water treatment costs and impedes the development of marine economies. Numerous high-sensitivity sensors, based on novel materials and advanced technologies, have been developed and widely implemented for the determination of nutrients in aquatic systems [7]. Despite the significance of ammonium in marine ecosystems, there are relatively few studies documenting its concentrations in global waters compared to other inorganic macronutrients like nitrate and nitrite. Challenges in accurately measuring ammonium concentrations stem from potential contamination during sampling and analysis, the absence of matrix-certified reference materials, and the inherent variability of ammonium species, which typically exist at low concentrations. The residence time of ammonium in surface waters is typically in the order of a few hours or even less [8]. Furthermore, the ammonium inventory in the ocean is approximately 1000-fold lower than that of nitrate, with concentrations generally ranging from zero to several hundred nanomolar. Average concentrations in the sunlit euphotic zone and the dark aphotic zone are about 300 nmol/L and 10 nmol/L, respectively [9,10]. As a result, there is an urgent need for a simple, convenient, and highly sensitive automated method to quantify ammonium in seawater, one that does not require additional enrichment or matrix separation, allowing analysis to be performed at the sampling site rather than a laboratory.

Compared with the commonly used indophenol blue (IPB) spectrophotometric method for ammonium measurement in seawater, fluorescence methods based on the reaction between ammonium and the derivatization reagent *o*-phthalaldehyde (OPA) offer 10- to 100-fold higher sensitivity, even without additional technical enhancements [2]. The fluorescence reaction using OPA was first reported in 1956, and in 1978, a modified version involving OPA and nucleophiles was applied to determine ammonium in seawater for the first time [11]. Since then, research has focused on improving both selectivity and sensitivity. The modern fluorescence method, based on the OPA–sulfite–NH_3_ reaction, includes the addition of formaldehyde to form a stable sulfite solution [12,13], and has become widely recognized for selectively measuring ammonium in aquatic environments. The product generated by the OPA–sulfite–NH_3_ derivatization reaction is 1-sulfonatoisoindole, with excitation and emission wavelengths at approximately λ_ex_ = 365 nm and λ_em_ = 425 nm, respectively. To further enhance sensitivity, enrichment techniques have emerged as an effective strategy. For instance, Zhu et al. [14] developed a sensitive flow-batch system that combined OPA-based fluorescence detection with solid-phase extraction (SPE) for ultra-trace ammonium determination in seawater. This method efficiently extracted fluorescent compounds onto a hydrophilic–lipophilic balance cartridge, which separated the analyte complex from the seawater matrix and enriched ammonium concentrations. The detection limits for this system in land-based and shipboard laboratories were 0.7 and 1.2 nmol/L, respectively. Other enrichment techniques, such as chromatographic separation, gas diffusion, and purge-and-trap methods, have also been shown to effectively enhance sensitivity [15]. However, while these enrichment methods enable nanomolar scale determination of ammonium, they increase system complexity and introduce potential failure risks. Moreover, the additional steps required for enrichment and extraction reduce the sample throughput, making these methods unsuitable for in situ underwater applications. Alternatively, two strategies can enhance the sensitivity of direct OPA fluorescence methods for ammonium detection in seawater without requiring sample enrichment. The first approach involves optimizing the chemical reactions in the fluorescence method, such as modifying the benzene ring of OPA to explore alternative derivatization reagents. This modification shifts the maximum excitation and emission wavelengths of the fluorescent products into the visible range, making it easier to select a suitable light source and improve sensitivity [16,17]. The second approach focuses on improving the fluorescence detector, the core component of the method, by developing more sensitive light detection components or optimizing the structural design of the flow cell. This can help reduce the detection limit and increase sensitivity. Fluorescence detectors used in the field or for in situ applications are typically compact and low-power, often designed to be portable. While such designs improve convenience, they require compromises in power and optical structure, which can reduce sensitivity. Although various portable fluorescence detectors have been developed as alternatives to commercial models [18], there remains significant potential for improving the sensitivity of these portable systems.

The combination of fluorometry and flow-analysis technology has enabled the on-site measurement of ammonium in water. Various flow techniques, including flow injection analysis [19,20], sequential injection analysis [21], continuous flow analysis [13], and flow-batch [18,22], have been integrated with the OPA fluorescence method to automate ammonium measurement in seawater. Over the past decade, a new flow technique known as lab-in-syringe (LIS) has gained widespread attention and application. LIS is considered as a hybrid of flow-batch analysis and sequential injection analysis. It has proven to be a versatile tool for automating sample preparation, particularly in liquid-phase microextraction [23]. In recent years, LIS has been applied to ammonium determination in water samples. For example, a syringe-pump-based analyzer using the IPB spectrophotometric method was developed for online ammonium analysis in coastal waters [24]. Additionally, an online flow method for ammonium detection in natural waters was established by combining the alternative derivatization reagent, 4,5-dimethoxyphthalaldehyde, with the LIS technique [25]. Furthermore, indirect fluorescence applications that integrate LIS with dual-headspace gas–liquid microextraction have also been introduced for ammonium determination in natural water samples [26]. These applications highlight LIS technology’s suitability for automating ammonium measurements in water bodies. It is anticipated that combining a highly sensitive portable fluorescence detector with LIS technology will broaden its applications.

To establish a simple, low-cost, and highly sensitive system for in situ trace ammonium measurement in seawater, a light-emitting-diode-induced fluorescence detector with a confocal structure employing a new photodetector *AccuOpt* 2000 was integrated with the LIS technology in our work. This direct fluorescence method eliminated the need for additional enrichment or extraction steps. After careful optimization of various parameters, the seawater matrix effect and performance characteristics were thoroughly investigated. Compared with our previous work and other reported direct fluorescence methods, the sensitivity of this method was significantly improved. The proposed method was successfully employed to measure the ammonium profile in the South China Sea and surface ammonium concentration in coastal seawater.

## 2. Results and Discussion

### 2.1. Parameter Optimization

**Figure 1:**
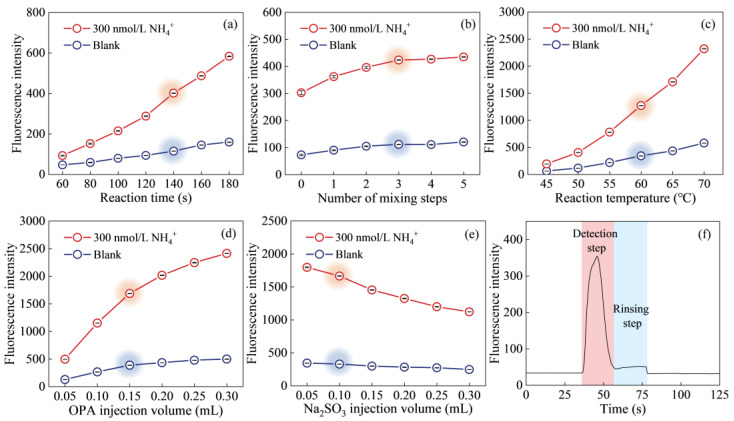
Effect of various parameters on fluorescence intensity (*n* = 3). (**a**) Reaction time, (**b**) number of mixing steps, (**c**) reaction temperature, (**d**) injection volume of OPA, (**e**) injection volume of Na_2_SO_3_, and (**f**) typical output signal for sample measurement.

To optimize the conditions for ammonium determination using the proposed flow system, a univariate experimental design was employed to systematically evaluate key parameters, including reaction time, number of mixing steps, reaction temperature, and reagent concentration in the final solution. Optimization experiments were conducted using both a blank and a 300 nmol/L ammonium standard solution, with triplicate measurements under varying conditions. During the optimization of each parameter, all other parameters were held constant at their previously determined optimal values to isolate the effects of the variable being tested. The results are presented in Figure 1.

The excitation and emission spectra of the reaction product, along with fluorescence intensities as a function of reaction time at different temperatures, are reported in our previous study [14]. It was found that the fluorescence reaction required 3–4 h to reach equilibrium at ambient temperature. Reaction time and temperature were identified as crucial factors influencing the sensitivity of the method. Furthermore, it is important to note that the chemical reaction was performed under non-equilibrium conditions within the flow system. Reaction time affects the extent of the reaction, and consequently, the fluorescence signal intensity. Extending the reaction time can enhance sensitivity. The effect of reaction time, ranging from 60 to180 s, was tested, as shown in Figure 1a. The net fluorescence signal increased significantly throughout the entire time range, with a gradual rise until it plateaued at 140 s. Considering the balance between peak height, blank levels, and sample throughput, the optimal reaction time was chosen as 140 s.

The degree of mixing between the sample and reagents also plays a crucial role in the magnitude and stability of the fluorescence signal. To achieve better mixing, a 2 mL microfluidic reservoir was added as a secondary mixing chamber in the flow system. The mixed solution was circulated between the syringe and the mixing chamber several times to ensure thorough homogenization. The impact of the number of mixing steps was tested by varying the number of steps from 0 to 5, as shown in Figure 1b. As the number of mixing steps increased, the net fluorescence signal also increased, suggesting that additional mixing steps enhanced reaction sensitivity. However, when three mixing steps were applied, the increase in fluorescence signal became negligible. Therefore, three mixing times were chosen as the optimal value for further optimization.

Figure 1c illustrates the effect of reaction temperature. Similar to the investigation of reaction time, the experimental results revealed that as the reaction temperature increased from 45 to 70 °C, the net fluorescence signal consistently improved. This enhancement was attributed to the accelerated reaction rate at higher temperatures. However, it is important to note that the blank signal also increases with temperature. Moreover, at elevated temperatures, equipment power consumption rises, and the formation of bubbles in the tubing may occur, potentially affecting the measurement accuracy. Therefore, the optimal reaction temperature was determined to be 60 °C.

The effect of OPA concentration in the mixed solution was examined by varying the volume of OPA injected into the syringe. The results, showing the impact OPA injection volumes ranging from 0.05 to 0.30 mL on the fluorescence signal intensity, are presented in Figure 1d. The net fluorescence signal increased across the entire volume range. However, considering the linear response range of the high-sensitivity fluorescence detector and the reagent consumption, an OPA injection volume of 0.15 mL (resulting in a OPA concentration of 2.78 mmol/L in the mixed solution) was chosen for this study. Similarly, the volume of Na_2_SO_3_ injected into the system was varied from 0.05 to 0.30 mL, and the corresponding results are shown in Figure 1e. The net fluorescence signal decreased across the entire Na_2_SO_3_ injection volume range. A similar effect was observed in our previous study [14]. As Na_2_SO_3_ is a nucleophilic reagent that plays a crucial role in the fluorescence reaction system, a very small injection volume could introduce measurement uncertainty, affecting the precision of the results. Based on these considerations, an Na_2_SO_3_ injection volume of 0.10 mL, corresponding to a concentration of 0.74 mmol/L in the mixed solution, was selected. Under the chosen optimal conditions, the typical output signal for sample measurements is shown in Figure 1f. As seen in the figure, variations in the signal were observed during both the peak and washing process as the mixed solution entered the fluorescence detector.

### 2.2. Seawater Matrix Effect

**Figure 2:**
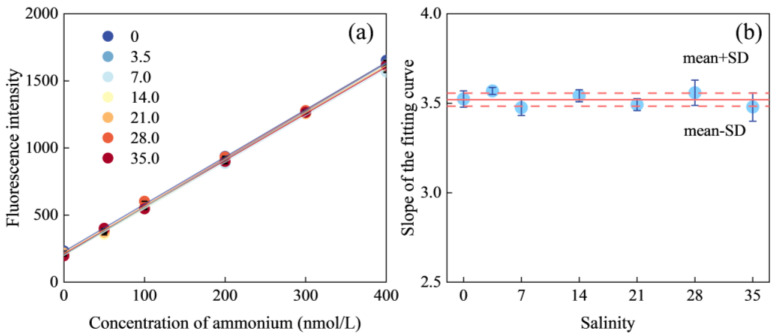
Investigation of seawater matrix effects. (**a**) Fitting curves for ammonium in samples with different seawater matrices and (**b**) relationship between the slope of the fitting curves and salinity.

Seawater, in contrast to freshwater, is characterized by higher salinity, higher pH, higher ionic strength, and a carbonate buffering capacity. These differences can influence the chemical reaction rates or alter reaction equilibria, which may ultimately affect the accuracy of quantitative results. The matrix effects of seawater, including, but not limited to, the effects of salinity, refractive index, and ionic strength, are well-documented and have been extensively studied, especially in the context of flow-analysis methods used for nutrient measurements in seawater. To evaluate the seawater matrix effect, aged low-nutrient seawater (LNSW) collected from the surface waters of the Indian Ocean was diluted with ultrapure water to simulate natural seawater with varying matrix compositions. This dilution process resulted in a series of seawater matrices with varying salinity levels of 0, 3.5, 7, 14, 21, 28, and 35. To minimize potential measurement errors caused by using single-concentration samples, standard ammonium solutions were spiked into each seawater matrix, creating a set of samples with multiple concentrations. The ammonium concentration range and concentration points were kept consistent across all seawater matrix conditions. Subsequently, the concentration results obtained under each seawater matrix condition were subjected to linear regression analysis. The degree of matrix interference and its trend were characterized by comparing the slopes of the fitting curves. Figure 2 displays the fitting curves for different salinity levels and illustrates the relationship between the slope of the fitting curves and salinity. The seven fitting curves exhibited nearly identical slopes. Deviations across seawater matrices remained below ±3.69%, confirming negligible matrix effects. Additionally, the carryover effect, which refers to the transport of an analyte from one sample to the next by the analytical system, was also re-evaluated in this study. Following the methodology outlined in previous research [27], the carryover effect was investigated by first measuring a high-concentration ammonium standard of 300 nmol/L, followed by two equal low-concentration ammonium standards of 50 nmol/L. The carryover coefficient was determined to be 0.06%, indicating the negligible influence of the carryover effect.

### 2.3. Analytical Figures of Merit

**Figure 3:**
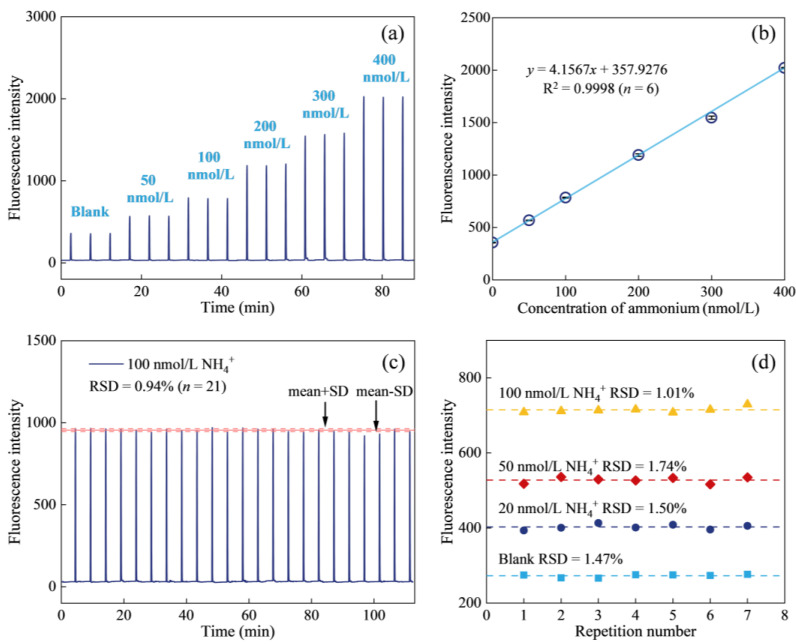
Analytical performance of the proposed ammonium measurement method for seawater. (**a**) Typical output signals of the calibration curve, (**b**) calibration curve, (**c**) reproducibility of the method for repeated measurements of a 100 nmol/L ammonium standard (*n* = 21), and (**d**) reproducibility for repeated measurements of ammonium samples at various concentrations (*n* = 7).

The performance of the method was evaluated based on the calibration curve, limit of detection (LOD), reproducibility, and sample throughput. The typical signal outputs of the calibration curve (*n* = 3) for ammonium concentrations up to 400 nmol/L are shown in Figure 3a. The regression equation obtained was y = 4.1567x + 357.9276, with R^2^ = 0.9998 (*n* = 6), where y was the fluorescence intensity and x was the concentration of ammonium (Figure 3b). The LOD, estimated as three times the standard deviation of the blank (*n* = 11), was 0.90 nmol/L, which was sufficiently low for ammonium determination in most open ocean waters. Reproducibility was assessed by repeating the measuring a standard sample with a concentration of 100 nmol/L ammonium. The results, shown in Figure 3c, demonstrated a relative standard deviation (RSD) of 0.94% (*n* = 21), indicating stable performance over extended measurement periods. To further assess repeatability across different concentrations, ammonium standard solutions ranging from 0 to 100 nmol/L were prepared, and each concentration was measured in seven replicates. The RSDs for replicate measurements of 0, 20, 50, and 100 nmol/L ammonium were 1.47%, 1.50%, 1.74%, and 1.01%, respectively (*n* = 7), confirming the method’s high precision (Figure 3d). The sample throughput was approximately 12 samples per hour.

A comparison of the analytical performance of the proposed method with other direct fluorescence methods for ammonium measurement in seawater, including the modern OPA–sulfite–NH_3_ reaction with formaldehyde addition post-2008 [2,13], is summarized in Table 1. The proposed method demonstrated several advantages, including high sensitivity, low LOD, low cost, and excellent repeatability. Compared with the recently reported similar flow systems [25], the proposed method achieved a nearly 10-fold improvement in sensitivity.

### 2.4. Validation of the Method

**Figure 4:**
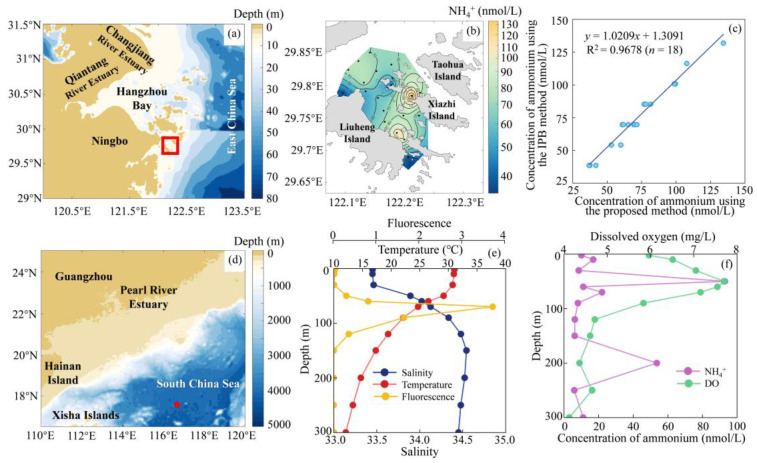
Validation and application of the proposed method. (**a**) Sample collection area in coastal seawater, (**b**) ammonium distribution in surface waters near Liuheng Island, Zhejiang Province, China (April 2024), (**c**) comparison of results between the proposed method and the IPB reference method, (**d**) location of ammonium profile measurements in the South China Sea (July 2024), (**e**) vertical profiles of temperature, salinity, and fluorescence intensity spanning 0–300 m, and (**f**) vertical distribution of ammonium and dissolved oxygen concentration in the upper 300 m.

The well-established IPB method, based on the Berthelot reaction, is the most widely used colorimetric technique for determining ammonium in seawater and has been adopted as the standard method by the U.S. Environmental Protection Agency [32] and other national testing agencies. To evaluate the accuracy of the proposed method, a comparison was made with the IPB reference method. Ammonium seawater samples collected from the Zhejiang coastal area were analyzed using both the IPB method and the proposed method. As shown in Figure 4c, the linear regression analysis between the two sets of results yielded a regression coefficient of 0.9678 (*n* = 18), with a slope of 1.0209, indicating excellent agreement between the two methods.

### 2.5. Field Application

Figure 4a shows the sample collection area of the coastal seawater near Liuheng Island, Zhejiang Province, China. The cruise conducted in April 2024 aimed to investigate and assess the marine environmental quality of the Zhejiang offshore region. The distribution of surface ammonium concentrations, ranging from 41.8 nmol/L to 134.4 nmol/L, across 20 investigation stations, is presented in Figure 4b. Overall, ammonium concentrations in the surface seawater of the study area were relatively low, likely due to the minimal human impact and wastewater discharges in this region. Additionally, the depletion of nutrients in the surface seawater, driven by photosynthetic processes, may also have contributed to this observation. The proposed method was further applied to determine the ammonium profile in the South China Sea in July 2024 (Figure 4d). The vertical distribution of ammonium in the upper 300 m at an investigation station (116.5° E, 17.3° N) is depicted in Figure 4f. Vertical profiles of temperature, salinity, fluorescence, and dissolved oxygen concentration from 0 to 300 m were also measured and are shown in Figure 4e,f. Ammonium concentrations exhibited significant variation within the euphotic zone, with two peaks observed in the upper 300 m. A subsurface ammonium maximum, of approximately 93 nmol/L, was found at around 50 m, coinciding with the subsurface maximum of dissolved oxygen and the thermocline. This distribution of ammonium is commonly attributed to phytoplankton uptake in the euphotic zone, ammonium regeneration from sinking phytoplankton biomass near the base of the euphotic zone via ammonification, and ammonium consumption through oxidation in the mesopelagic zone.

## 3. Experimental

### 3.1. Chemicals and Reagents

All chemicals used in this study were of analytical grade and were purchased from Thermo Fisher Scientific Inc. (Waltham, MA, USA), unless stated otherwise. Solutions were prepared using ultrapure water obtained from a pure water purification system (Millipore, Burlington, MA, USA), with a resistivity greater than 18.2 MΩ·cm at 25 °C. High-density polyethylene (HDPE, Nalgene, New York, NY, USA) bottles were used for storing reagents, standard solutions, and water samples. All containers and bottles were pre-cleaned by soaking in 3 mol/L HCl solution overnight, followed by thorough rinsing with ultrapure water at least 3 times before use. To minimize the risk of ammonia contamination from external air sources in reagents, this study further improved the air inlet clean-up devices for reagent and sample bottles, as reported in our previous study [14]. In addition to the acidic silica gel, the device now included fiberglass and a needle filter. This segmented connection design effectively absorbed ammonia from the air, preventing particulate matter from entering the reagent bottle, and ensuring that silica gel did not spill into the bottles, making it more suitable for online analysis applications.

The OPA stock solution (25.0 mmol/L) was prepared by dissolving 0.8383 g of OPA in 50 mL of methanol, then diluting it to 250 mL with ultrapure water. The solution was stored in an amber bottle to prevent photodegradation. A 10.0 mmol/L sodium sulfite (Na_2_SO_3_) stock solution was prepared by dissolving 0.3151 g of Na_2_SO_3_ in 250 mL ultrapure water, and adding 0.1 mL formaldehyde (HCHO, Lingfeng Chemical Reagent Co., Shanghai, China) to prevent oxidation and reduce potential interference. A 15 g/L sodium tetraborate decahydrate (Na_2_B_4_O_7_) buffer solution was obtained by dissolving 3.75 g Na_2_B_4_O_7_·10H_2_O in 250 mL ultrapure water. The ammonium stock solution with a concentration of 100 mmol/L was prepared by dissolving 1.07 g of ammonium chloride (NH_4_Cl), which was previously dried at 105 °C for 2 h, in 200 mL ultrapure water. All solutions were stored at temperature below 4 °C, and daily working standard solutions were prepared by diluting the stock solution as needed.

### 3.2. Apparatus

Light-emitting diodes (LEDs) have become the primary light source in portable fluorescence detectors, replacing high-power xenon lamps typically used in benchtop instruments. This shift is due to their advantages, including long lifetime, high stability, compact size, low cost, and availability across a wide range of wavelengths, from deep ultraviolet (UV) to near-infrared. However, the light emitted by LEDs generally has a wide divergence angle, owing to its area source and incoherent nature. This makes it challenging to focus the LED light into a small spot that matches the inner diameter of a fused-silica capillary [33]. To address this issue, a simple and compact configuration design for the excitation components, including the LED, excitation filter, pinhole, and capillary, were developed to minimize the distance between the LED source and the detection cell. This adjustment ensured that the maximum amount of LED light reached the detection cell. As a result, this modification led to a 3.5-fold increase in the signal-to-noise ratio (SNR) compared with previous studies [33]. Recently, a novel photodiode-based weak-light detector, *AccuOpt* 2000, which offers high sensitivity was developed by the Dalian Institute of Chemical Physics, Chinese Academy of Sciences. The *AccuOpt* 2000 features a dynamic range exceeding five orders of magnitude, with a maximum output voltage of 10 V at a ±12 V power supply. Notably, the *AccuOpt* 2000 does not require high polarization voltage, is immune to electromagnetic interference, and operates effectively under ambient indoor lighting conditions without performance degradation [34]. These advantages make it an ideal replacement for traditional photomultiplier tubes in fluorescence detection.

**Figure 5:**
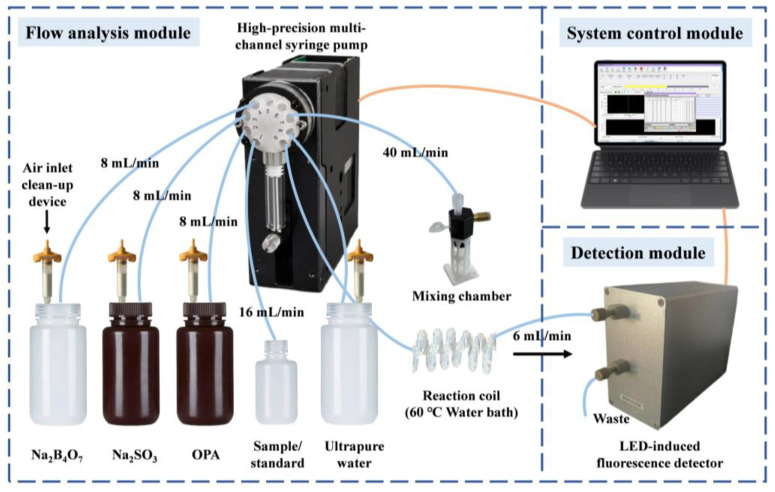
Schematic diagram of the proposed flow system for ammonium determination in seawater.

The flow system arrangement is shown in Figure 5. The system consisted of the following components: a high-precision multi-channel syringe pump (Nanjing Runze Fluid Control Equipment Co., Ltd., Nanjing, China) equipped with a 9-port selection valve and a gastight 2.5 mL borosilicate glass ILS micro syringe (Innovative Labor Systeme GmbH, Ilmenau, Germany), a thermostatic water bath (Shanghai Jing Hong Laboratory Instrument Co., Ltd., Shanghai, China), a mixing chamber (Fluidic Lab, Shanghai, China), and a fluorescence detection module (Dalian Scien & Tech Instrument Inc., Dalian, China). This fluorescence detection module featured a custom-designed LED-induced fluorescence detector specifically for ammonium detection, using a 365 nm ultraviolet UV-LED (Shenzhen Yuanchuang Electronic Co., Shenzhen, China) as the excitation light source. The module included a silicon photoelectric detector assembly, the *AccuOpt* 2000, and had a compact design with a fully solid-state optical path. The detection cell, made of quartz, had a volume of 28 µL and could withstand pressures up to 2 MPa. System control and data acquisition were managed through custom LabVIEW 8.20 (NI, Austin, TX, USA) software. The entire fluidic manifold was constructed using PTFE tubing (0.8 mm id.) and standard 1/4-28 flangeless PEEK fittings (VICI, Valco Instruments Co., Houston, TX, USA).

### 3.3. Analytical Procedure

The operation program of the proposed system is outlined in Table S1 and described as follows: First, 0.50 mL of the sample, 0.15 mL of OPA, 0.10 mL of Na_2_SO_3_, 0.10 mL of Na_2_B_4_O_7_, and an additional 0.50 mL of the same sample were sequentially introduced into the syringe via the selection valve, where they underwent an initial mixing step. The sample was added in two portions to facilitate more uniform mixing with the reagents. The mixed solution was then transferred to the mixing chamber and drawn back into the syringe. This process was repeated several times to ensure thorough mixing. The mixed solution was subsequently transferred into a pre-assembled reaction coil and heated at 60 °C for 140 s. Finally, the reacted solution was injected into the flow cell of the custom-designed LED-induced fluorescence detector, where the fluorescent signal was captured and recorded. Before and after analysis, each sample was washed through the entire flow path with ultrapure water.

### 3.4. Sample Collection

Profiled seawater samples were collected from various depths at a fixed station in the South China Sea (116.5° E, 17.3° N) using a 12-bottle CTD rosette sampler system (SBE-911/917 Plus CTD, Seabird Co., Bellevue, WA, USA) during a cruise in July 2024. Estuarine and coastal seawater samples, collected from the surface of Hangzhou Bay (Zhejiang Province, China) near Liuheng Island in April 2024, were used for validation experiments. These coastal-area seawater samples were filtered through a 0.45 μm membrane filter and then stored in acid-washed HDPE bottles at −20 °C.

## 4. Conclusions

In summary, a highly sensitive automated flow system utilizing direct fluorescence was established for trace ammonium determination in seawater, eliminating the need for additional enrichment or extraction steps. Compared with similar flow-analysis systems for ammonium detection in seawater, this system exhibited several advantages, including high sensitivity, adherence to a classical chemical reaction framework, and considerable potential for in situ/on-site applications. It had the analytical characteristics of low LOD (0.90 nmol/L), good precision (RSD < 1.8%, *n* = 7), stable performance over extended measurement periods (RSD = 0.94%, *n* = 21, 100 nmol/L), extended linear range (up to 400 nmol/L), and high sample throughput (12/h). In addition, the seawater matrix effect and carryover effect were negligible. The results obtained using the proposed method showed excellent agreement with those from the classical IPB spectrometric method. The robust analytical performance and successful field applications collectively validate the feasibility for trace ammonium monitoring in marine environments. Furthermore, the design of the direct fluorescence method simplifies the system, making it a promising and efficient tool for high spatial and temporal resolution in situ ammonium measurements in seawater.

## Figures and Tables

**Table 1:** Summary of the analytical performance of other direct fluorescence methods for ammonium measurement in seawater since 2008.

Year	Ref.	Chemistry	Technique	LOD (nmol/L)	Linear Range (μmol/L)	Precision (% RSD)
2008	[13]	OPA–sulfite with HCHO	CFA or FIA	1.1	0.1–12	2.2 (200 nmol/L); 6.7 (1 nmol/L)
2011	[28]	OPA–sulfite	CFA	<5	0.05–25	1–4 (5 nmol/L-25 μmol/L)
2011	[29]	OPA–sulfite	Multi-pumping flow analysis	13	Up to 16	<2 (5 μmol/L)
2011	[22]	OPA–sulfite with HCHO	Modified SIA (termed as ABA)	1	0.005–25	0.6 (200 nmol/L)
2013	[18]	OPA–sulfite with HCHO	Modified ABA with a portable detector	10	0.05–10	3 (2 μmol/L)
2015	[30]	OPA–sulfite with HCHO	rFIA	1.1	Up to 1	2.4 (1 μmol/L)
2023	[31]	NDA–sulfite	Merging-zone flow injection	45	0.045–6	3.68 (1.5 μmol/L)
2024	[25]	M_2_OPA–sulfite with HCHO	LIS	9.04 (25 °C) 15.11 (50 °C)	0.015–0.6	1.21 (25 °C, 50 nmol/L) 3.26 (50 °C, 50 nmol/L)
This method		OPA–sulfite with HCHO	LIS	0.9	Up to 0.4	0.94 (100 nmol/L)

Note: CFA, continuous flow analysis; FIA, flow injection analysis; SIA, sequential injection analysis; rFIA, reverse flow injection analysis; ABA, autonomous batch analyzer; LIS, lab-in-syringe; NDA, naphthalene-2,3-dicarboxaldehyde.

## Data Availability

The original contributions presented in this study are included in the article/Supplementary Material. Further inquiries can be directed to the corresponding authors.

## Supplementary Materials

The following supporting information can be downloaded at: https://www.mdpi.com/article/10.3390/molecules30061288/s1, Table S1: Operational program of the proposed system for ammonium determination in seawater samples.

## Abbreviations

The following abbreviations are used in this manuscript:

IPB	Indophenol blue
OPA	*o*-phthalaldehyde
SPE	Solid-phase extraction
LIS	Lab-in-syringe
LED	Light-emitting diode
LNSW	Low-nutrient seawater
CFA	Continuous flow analysis
FIA	Flow injection analysis
SIA	Sequential injection analysis
rFIA	Reverse flow injection analysis
ABA	Autonomous batch analyzer
NDA	Naphthalene-2,3-dicarboxaldehyde
