# Supplementary material for: Automated Determination of Ammonium at Nanomolar Levels in Seawater by Coupling Lab-in-Syringe with Highly Sensitive Light-Emitting-Diode-Induced Fluorescence Detection
Source: Molecules. 2025 Mar 13;30(6):1288. doi: 10.3390/molecules30061288 (PMC11945248; doi:10.3390/molecules30061288)
Supplement: Supplementary file 1 [file molecules-30-01288-s001.zip › molecules-3494661-supplementary.pdf]

Table S1 Operational program of the proposed system for ammonium determination in seawater samples

Step	Multi-channel syringe pump Rate (mL/min)	Multi-channel syringe pump Valve position	Duration time (s)	Comments
1	40	8	6	Washing the entire flow path with ultrapure water
2	40	7	6	
3	40	7	6	
4	40	9	6	
5-8				Repeating steps 1-4 for thorough cleaning
9	40	1	5	Rinsing the entire flow path with the water sample
10	40	7	5	
11	40	7	5	
12	40	9	5	
12-16				Repeating steps 9-12
17	16	1	6	Aspirating 0.50 mL of the sample into the syringe
18	8	2	5	Aspirating 0.15 mL of OPA into the syringe
19	8	3	5	Aspirating 0.10 mL of Na_2SO_3 into the syringe
20	8	4	5	Aspirating 0.10 mL of $Na_2B_4O_7$ into the syringe
21	16	1	6	Aspirating an additional 0.50 mL of the same sample into the syringe
22	40	7	4	Transferring the mixed solution from the syringe to the mixing chamber and drawing it back into the syringe
23	40	7	4	
24-27				Repeating steps 22 and 23 twice to ensure proper mixing
28	40	9	4	Transferring the mixed solution into the pre-assembled reaction coil and heating it at 60 °C
29	40	8	140	Forming fluorescent compounds
30	6	9	33	Loading the reacted solution into the detector for analysis
